# Micropulse Laser Trabeculoplasty in Glaucoma: Efficacy and Safety in a Clinical Cohort

**DOI:** 10.3390/medicina61122129

**Published:** 2025-11-28

**Authors:** Flaviu Ionut Bodea, Cristina Ariadna Nicula, Delia Mirela Tit, Andrei-Flavius Radu, Ruxandra Cristina Marin, Gabriela S. Bungau

**Affiliations:** 1 Doctoral School of Biological and Biomedical Sciences, University of Oradea, 410087 Oradea, Romania; dr.flaviu.bodea@gmail.com (F.I.B.); marin.ruxandracristina@student.uoradea.ro (R.C.M.); gbungau@uoradea.ro (G.S.B.); 2 Department of Pharmacy, Faculty of Medicine and Pharmacy, University of Oradea, 410028 Oradea, Romania; 3 Department of Psycho-Neurosciences and Recovery, Faculty of Medicine and Pharmacy, University of Oradea, 410073 Oradea, Romania; andreiflavius.radu@uoradea.ro; 4 Department of Pharmacology, Clinical Pharmacology and Pharmacotherapy, Faculty of Medicine, “Carol Davila” University of Medicine and Pharmacy, 050474 Bucharest, Romania

**Keywords:** micropulse laser trabeculoplasty, open-angle glaucoma, intraocular pressure, non-invasive treatment, laser therapy

## Abstract

*Background and Objectives*: Glaucoma is a leading cause of irreversible blindness, and lowering intraocular pressure (IOP) is the only proven strategy to slow disease progression. We evaluated the clinical efficacy and safety of Micropulse Laser Trabeculoplasty (MLT) in patients with open-angle glaucoma and ocular hypertension, focusing on IOP control, visual function, and adverse events. *Materials and Methods*: This longitudinal, real-world cohort included 80 patients (132 eyes) treated with MLT between 2018 and 2025 at the Ophthalmology Clinic of the County Emergency Hospital, Bihor. Micropulse laser trabeculoplasty was applied over 360°, except in selected cases (90–300°), depending on anatomical or clinical factors. Outcomes included IOP by Goldmann and non-contact tonometry, best-corrected visual acuity (BCVA), refraction, and safety events. Pre-/post comparisons used paired tests and McNemar’s exact test where appropriate. *Results*: IOP decreased from 18.15 ± 5.02 to 15.57 ± 3.78 mmHg at 3 months (mean reduction: 2.58 mmHg, *p* < 0.001), confirmed by GEE adjusted for age, sex, and eye laterality. The proportion of eyes within target ranges increased significantly (IOP ≤ 18 mmHg and ≤21 mmHg; *p* = 0.0014 and *p* = 0.0023, respectively). A total of 31.1% of eyes achieved ≥ 20% IOP reduction, and 31.8% had an absolute decrease > 3 mmHg. BCVA and refraction remained stable (*p* > 0.05). No major complications or IOP spikes > 5 mmHg occurred; transient, self-limited events were uncommon. *Conclusions*: MLT was associated with a safe and clinically relevant reduction in IOP in patients with open-angle glaucoma and ocular hypertension, with stable visual function and minimal adverse effects observed. These results suggest that MLT may be a useful option for IOP management; however, the lack of a control group limits causal interpretation. Further controlled studies are warranted to confirm these findings.

## 1. Introduction

Glaucoma represents a chronic, progressive optic neuropathy characterized by irreversible loss of retinal ganglion cells and visual field defects [1], remaining one of the leading causes of blindness worldwide [2]. As life expectancy increases worldwide, the prevalence of glaucoma is projected to rise substantially, with estimates suggesting that more than 110 million people will be affected by 2040 [3]. This global burden underscores the need for therapeutic strategies that are both effective and sustainable over a lifetime of management. The disease’s asymptomatic course in early stages and its chronic nature require long-term treatment adherence and monitoring, making approaches that minimize patient burden and preserve ocular surface health particularly valuable [4]. Advances in laser and pharmacologic technologies now reflect a shift toward minimally invasive, patient-centered interventions that can maintain stable intraocular pressure (IOP) while reducing treatment complexity and improving quality of life [5].

Despite the continuous development of neuroprotective strategies, reduction in IOP remains the only evidence-based intervention proven to delay or prevent disease progression. In clinical practice this is achieved with medications, laser therapies, or surgery. Laser trabeculoplasty has long been an important non-invasive intervention for open-angle glaucoma management, and it represents an important intermediate step between medical and surgical treatment, aiming to improve aqueous humor outflow through the trabecular meshwork (TM) without altering the integrity of ocular structures. Beginning with argon laser trabeculoplasty (ALT) in 1979 and later selective laser trabeculoplasty (SLT) in the 1990s, laser treatments have offered IOP-lowering efficacy comparable to medications, with SLT having the advantage of less tissue damage and repeatability [6]. While ALT provided effective IOP reduction, it induced structural scarring, limiting repeatability. On the other hand, SLT, employing a Q-switched 532 nm Nd:YAG laser, selectively targeted pigmented TM cells, minimizing collateral damage but occasionally producing inflammation or transient IOP spikes [7].

Topical pharmacologic therapy is the first-line approach in most cases; however, poor adherence, reported to be as low as 60% and ocular surface toxicity from preservatives frequently limit its long-term effectiveness [8], and may lead to medication discontinuation [9]. In this context, there is growing emphasis on treatment strategies that combine efficacy with safety, comfort, and sustainability. The therapy paradigm has gradually changed due to advancements in laser technology, moving from destructive to subthreshold modalities that preserve tissue integrity while causing the trabecular meshwork to biologically activate. This transition reflects a broader evolution in glaucoma care toward individualized, minimally invasive, and repeatable approaches that can be seamlessly integrated into chronic disease management [10,11].

Consequently, the search for a safe, repeatable, and effective laser alternative has led to the development of micropulse laser trabeculoplasty (MLT), a newer innovation introduced in the mid-2000s that delivers laser energy in a subthreshold manner using a duty cycle (typically 15%) of very short laser pulses separated by brief rest intervals. This micropulse emission pattern allows thermal relaxation between pulses, limiting heat accumulation in the TM and minimizing collateral tissue damage, postoperative inflammation, and scarring compared with conventional continuous-wave laser applications. By avoiding coagulative damage, MLT preserves the integrity of the TM, making the procedure safer, less painful, and theoretically allowing for repetition if needed. Early studies have indicated that MLT is a promising option for patients with ocular hypertension or open-angle glaucoma (including pseudoexfoliative and pigmentary glaucoma), particularly in those who respond inadequately to medical therapy or seek to defer invasive surgery [12].

The biologic response to MLT involves cellular and biochemical activation rather than tissue ablation, with stimulation of TM endothelial and macrophage activity, upregulation of cytokines such as IL-1β and TNF-α, and remodeling of extracellular matrix pathways that enhance aqueous outflow while preserving TM morphology [13]. Experimental data confirm that sub-threshold stimulation up-regulates metalloproteinases and modulates oxidative stress pathways, offering a non-destructive mechanism of trabecular activation distinct from thermal ablation [14].

Clinically, MLT is typically performed using a 577 nm yellow diode laser, 200–300 µm spot size, 300 ms exposure, and 1000–1500 mW power applied over 180° or 360° of the angles under gonioscopy control. Several randomized and observational studies have confirmed its efficacy and safety under these parameters. A long-term series with 577 nm MLT reported sustained IOP reductions of 12–17% over 36–48 months, with acceptable retreatment rates and no significant adverse events [15]. Similarly, a cohort study of primary open-angle glaucoma (POAG) eyes demonstrated that MLT achieved durable IOP lowering with minimal anterior chamber inflammation and without peripheral anterior synechiae [16]. More recently, a systemic review reinforced these findings, emphasizing that MLT is both effective and safe as a primary or adjunctive therapy for glaucoma and ocular hypertension but that standardization of technique and longer follow-up are required to optimize outcomes [12].

Current evidence suggests that MLT achieves mean IOP reductions of 15–25% across open-angle glaucoma phenotypes, with higher baseline IOP predicting better response and consistent efficacy in pigmentary and pseudoexfoliative forms. Because the micro pulse technique prevents thermal injury, it can be safely applied in heavily pigmented TM without inducing inflammation or synechiae, and pseudophakia has not been shown to compromise outcomes [17]. MLT’s non-destructive nature and minimal postoperative inflammation profile also explain the excellent tolerability reported across multiple studies, where no significant IOP spikes (>5 mmHg), endothelial injury, or visual acuity changes were observed [18]. Moreover, clinical evidence indicates that MLT may allow partial reduction in topical medication burden while maintaining stable IOP control, a finding of increasing practical relevance for elderly and polytreated glaucoma patients [12].

The combination of repeatability, minimal discomfort, and lack of structural damage supports its inclusion as a viable intermediate option between pharmacologic and surgical management. Because of these properties, MLT aligns with the principles of modern glaucoma management, emphasizing tissue preservation, repeatability, and patient-centered care. It also holds potential for earlier therapeutic intervention, particularly in patients where adherence to pharmacologic therapy is suboptimal or ocular surface disease limits tolerance to multiple topical agents. The increasing amount of research and rapid clinical adoption of MLT underscore the need for empirical outcomes that verify its reproducibility, safety, and applicability across diverse patient populations [19].

This research evaluated the efficacy, safety, and clinical applicability of MLT in a real-world cohort of patients with open-angle glaucoma at various stages of severity and ocular hypertension. The analysis included POAG of varying severity, as well as pseudoexfoliative and pigmentary subtypes. The primary objective was to quantify IOP reduction over the short- to medium-term and assess its consistency across diagnostic categories. Secondary outcomes included changes in visual and refractive parameters and adverse events. The study also aimed to define MLT’s role within current glaucoma management, particularly relative to SLT. By applying standardized parameters and correlating clinical outcomes with recent evidence, this work provides an updated assessment of MLT as a reproducible and minimally invasive treatment option in contemporary glaucoma care.

## 2. Materials and Methods

### 2.1. Study Design and Patients

This study included patients diagnosed with open-angle glaucoma (primary, or secondary types: pseudoexfoliative and pigmentary) and ocular hypertension who underwent MLT between 2018 and 2025 at the Ophthalmology Department of the Emergency Clinical County Hospital, Bihor. The study was conducted on an outpatient basis. All patients were informed about the procedure, and written informed consent was obtained prior to inclusion. Inclusion criteria comprised open-angle glaucoma confirmed by gonioscopy (Shaffer grade 2–4) and ocular hypertension requiring IOP reduction. Exclusion criteria were angle-closure glaucoma, absolute or decompensated glaucoma, and neovascular glaucoma, where surgical intervention remains the only viable option.

The research followed the principles of the Declaration of Helsinki.

### 2.2. Laser Procedure

The MLT was performed using a slit-lamp-mounted, diode-pumped solid-state laser operating at 577 nm (Iridex system, Mountain View, CA, USA) in micropulse mode. The procedure was carried out with the patient seated at the slit-lamp, without mydriatic drops. In some cases, 2% pilocarpine was instilled preoperatively to improve visualization of the iridocorneal angle.

A dedicated gonioscopy treatment lens (MLT lens, Ocular Instruments, Bellevue, WA, USA) was used. This lens included a mirror for angle visualization, a rotational index ring, and a white marker to guide the treated trabecular area. Laser energy was applied tangentially and consecutively along the trabecular meshwork over approximately 360°, or partially (90–300°) as clinically indicated. A minimum of 120 laser spots (about 10 per clock hour) were applied, with parameters standardized as follows (Table 1).

### 2.3. Clinical Evaluation

All patients underwent a detailed ophthalmic examination both before and after the laser procedure. Each eye was examined individually using standardized instruments; however, all statistical analyses accounted for the non-independence of paired eyes through appropriate correlation-adjusted methods. The monitoring sheet included personal data, clinical diagnosis, ocular medication, and detailed examination findings. The main evaluations performed are summarized in Table 2.

### 2.4. Follow-Up and Data Processing

Clinical and paraclinical re-evaluation was performed 12 ± 2 weeks after treatment, with primary comparative data obtained at baseline and at the first post-treatment visit. Follow-up included assessment of visual acuity, refraction, pachymetry, intraocular pressure (via both Goldmann and non-contact tonometry), and gonioscopy evaluation of the iridocorneal angle morphology. Particular attention was paid to the presence or absence of peripheral anterior synechiae, trabecular scarring, or morphological changes in the angle.

Images (anterior segment OCT) of the treated areas were captured when necessary to document anatomical particularities. All data were recorded in an electronic database and statistically processed for descriptive and comparative analysis. The primary endpoint was the mean change in IOP (both air-puff and Goldmann) from baseline to the last available follow-up. Secondary outcomes included changes in BCVA, refractive parameters, and the incidence of postoperative complications. A clinically significant response was defined as an IOP reduction ≥ 20% from baseline (or there could also be a 3 mmHg decrease in intraocular pressure).

Goldmann applanation tonometry (GAT) was used as the primary IOP measurement for all analyses, as it is considered the clinical gold standard. Air-puff tonometry was recorded only as a secondary, supportive measurement and was not used to drive clinical or statistical conclusions. Although pachymetry values were documented, no pachymetry-based correction formula was applied to IOP, in line with evidence indicating poor reliability and lack of validation of such correction algorithms in glaucoma research [20,21].

Data were processed using Microsoft Excel and SPSS version 26.0. Continuous variables were expressed as mean ± standard deviation (SD) and compared using paired t-tests or repeated-measures ANOVA, while categorical data were analyzed with chi-square or Fisher’s exact tests. Statistical significance was set at *p* < 0.05.

The GEE model incorporated patient ID as the clustering variable, time (pre/post) as a within-subject factor, and eye laterality (OD Vs. OS) age (centered), and sex as covariates. To further assess whether the treatment effect varied across demographic subgroups, interaction terms (Moment × Sex and Moment × Age) were also tested in an extended model. Age was centered around the mean to improve model interpretability and reduce multicollinearity. Both eyes from the same patient were treated as correlated, rather than independent, observations within the GEE framework.

A Post Hoc power analysis was performed using the SPSS paired-sample t-test module. Based on the observed mean IOP reduction of 2.58 mmHg and a standard deviation of 5.02 mmHg, the effect size was estimated at Cohen’s d = 0.514. With a sample of 132 paired eyes and a two-tailed alpha of 0.05, the resulting statistical power exceeded 99.9%. This confirms that the study was sufficiently powered to detect clinically meaningful changes in IOP while minimizing the risk of Type II error.

## 3. Results

### 3.1. Patients’ Characteristics

A total of 80 patients (132 eyes) were included in the study. The mean age of the cohort was 64.0 ± 12.9 years (range 27–87), with a female predominance (62.9%). Bilateral MLT was performed in 65% of patients, while 35% received unilateral treatment. The distribution of age, gender, and eye laterality is summarized in Table 3.

In terms of glaucoma diagnosis, most eyes were diagnosed with POAG, with a smaller subset classified as advanced POAG. Other diagnoses included pigmentary, pseudoexfoliative, juvenile, and secondary glaucoma. On gonioscopic examination, most eyes had open angles (Shaffer grade 3 or 4). Gonioscopic particularities were observed in approximately one-third of eyes, the most frequent being a heavily pigmented trabecular meshwork. The clinical and gonioscopic characteristics of treated eyes are summarized in Table 4.

Ocular comorbidities or previous ocular surgeries were identified in approximately one-third of patients. The most frequent condition was pseudophakia, reflecting a history of cataract extraction in many cases. Other recorded findings included dry age-related macular degeneration, diabetic retinopathy, and prior retinal or glaucoma-related interventions. A detailed distribution of ocular comorbidities is provided in Table 5.

### 3.2. Pre-Treatment IOP and Medications

At baseline, the mean intraocular pressure was in the high teens for this cohort, reflecting that many patients were already on therapy (18.15 ± 5.02 mmHg). The majority of eyes were treated with topical intraocular pressure-lowering medications. The most prescribed drug classes included beta-blockers (primarily timolol), prostaglandin analogs (latanoprost, travoprost, or bimatoprost), and carbonic anhydrase inhibitors (such as dorzolamide and brinzolamide), often in fixed combinations. Alpha-agonists and acetazolamide were used less frequently. The summarized treatment categories are shown in Table 6.

### 3.3. Laser Treatment Parameters

**Figure 1:**
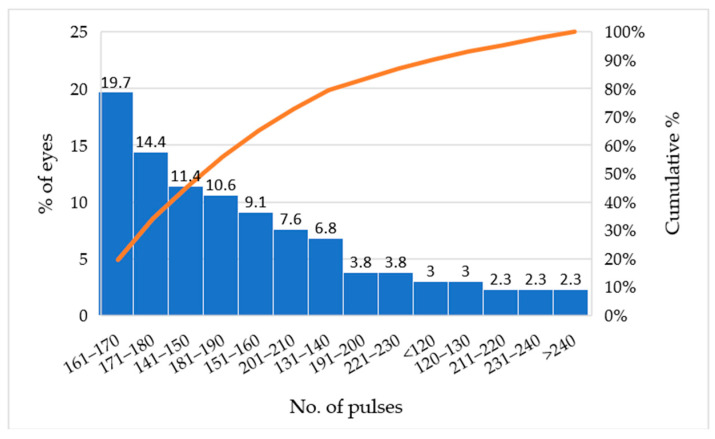
Distribution of eyes by number of pulses.

MLT was performed with fairly uniform settings across patients. In over 95% of treated eyes, the laser power was 1000 mW and the spot duration 300 ms with 15% duty cycle. A spot size of 300 µm was used in essentially all eyes (only 6 initial cases employed a 200 µm spot). Nearly all treatments (127 out of 132 eyes, 96.2%) were applied over 360° of the angles. Only a handful of eyes received a more limited sector treatment (one eye each treated over 90°, 180°, 300°, or 340° sectors, typically due to focal peripheral anterior synechiae or poor view in portions of the angle). The number of laser applications (spots) per eye varied depending on the angle circumference treated and any overlap, with a median in the range of 160–170 spots. The overall distribution of pulse counts is shown in Figure 1.

### 3.4. IOP Outcomes

**Figure 2:**
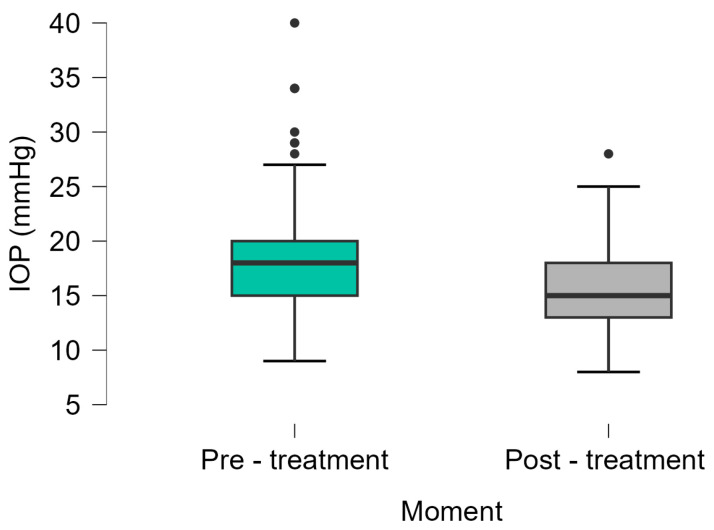
Evolution of IOP before and after micropulse laser trabeculoplasty. IOP, intraocular pressure.

Micropulse laser trabeculoplasty resulted in a statistically significant reduction in IOP, from 18.15 ± 5.02 mmHg pre-treatment to 15.57 ± 3.78 mmHg at 3-month follow-up (Figure 2).

To account for within-subject correlation and potential demographic confounders, IOP outcomes were analyzed using a generalized estimating equation (GEE) model. The analysis included patient ID as a clustering variable, and incorporated time (pre/post), sex, age (centered), and eye laterality (OD/OS) as covariates. In a subsequent extended model, interaction terms were added to assess whether the treatment effect varied by sex or age. The adjusted analysis confirmed a statistically significant average IOP reduction of 2.58 mmHg (*p* < 0.001). No significant effect was observed for age (*p* = 0.505), sex (*p* = 0.210), or eye laterality (*p* = 0.559). Moreover, interaction terms for Moment × Sex and Moment × Age were also non-significant (*p* = 0.999 and *p* = 0.153, respectively), indicating that the IOP-lowering effect of MLT was consistent across demographic subgroups (Table 7).

**Figure 3:**
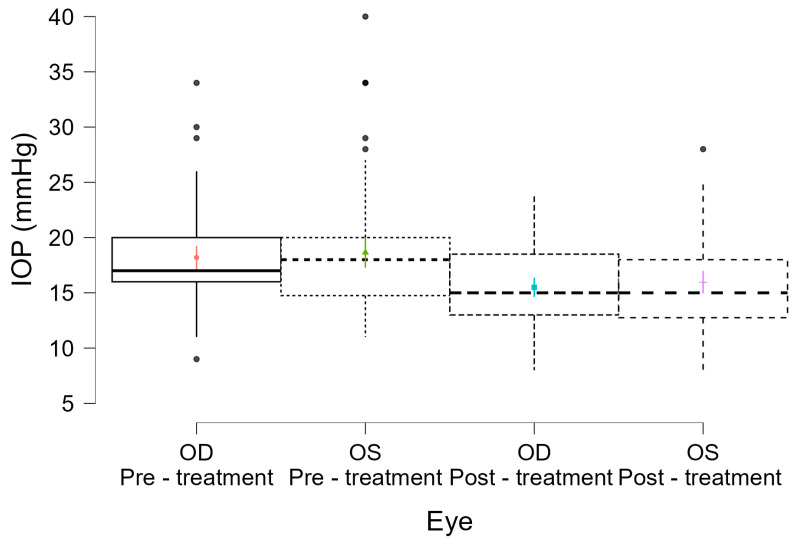
Distribution of intraocular pressure (IOP) before and after MLT, measured separately for right (OD) and left (OS) eyes.

This finding is further supported by Figure 3, which illustrates a comparable post-treatment IOP reduction for both right (OD) and left (OS) eyes, reinforcing the conclusion that eye laterality did not influence treatment efficacy.

Clinically, a single session of MLT effectively reduced IOP into target therapeutic ranges in most treated eyes. At follow-up, 76.5% of eyes achieved an IOP ≤ 18 mmHg, compared to 50.8% at baseline (*p* = 0.0014), and 95.4% achieved IOP ≤ 21 mmHg (vs. 86.3% pre-treatment, *p* = 0.0023). In addition, 31.1% of eyes experienced a ≥20% IOP reduction from baseline, and 31.8% had an absolute decrease > 3 mmHg (Table 8).

### 3.5. Visual Acuity and Refraction

Best-corrected visual acuity remained stable following MLT. The mean BCVA at baseline was 0.67 ± 0.33, compared to 0.70 ± 0.33 at follow-up (*p* = 0.55), with no statistically or clinically meaningful change observed. A GEE model accounting for inter-eye correlation and repeated measures confirmed the absence of a significant treatment effect on BCVA (mean difference = +0.0094; 95% CI: −0.013 to +0.032; *p* = 0.408).

Refractive parameters were similarly stable. The most common refractive profiles were myopia and myopic astigmatism. A statistically significant reduction in hyperopic astigmatism prevalence was observed (from 12.9% to 3.0%; *p* = 0.003), though the clinical impact of this change was minimal. Other refractive categories did not show significant shifts (Table 9).

### 3.6. Safety and Complications

No serious complications occurred during or after MLT. Most patients reported only mild discomfort during the procedure (9.8%), and transient conjunctival hyperemia or ocular ache occurred in 12.1% of eyes, typically resolving within 1–2 days, with or without topical NSAIDs.

A mild anterior chamber reaction (cells and flare) was noted in 6.8% of eyes at the first follow-up and resolved by one month with short-term anti-inflammatory therapy. Transient IOP spikes (≤4 mmHg) within the first hour post-laser were observed in 3.8% of cases and resolved spontaneously. Importantly, no cases of peripheral anterior synechiae, corneal complications, or vision-threatening events were recorded. Table 10 summarizes all observed adverse events and their outcomes.

## 4. Discussion

Glaucoma management continues to evolve toward treatment strategies that achieve sustained IOP control while minimizing structural damage and improving long-term tolerability. Within this therapeutic continuum, laser trabeculoplasty occupies a key position between topical pharmacotherapy and incisional surgery, providing an effective, repeatable, and minimally invasive method of enhancing trabecular outflow [12]. This study investigated the IOP-lowering efficacy and safety profile of micropulse laser trabeculoplasty (MLT) in eyes with open-angle glaucoma or ocular hypertension.

In our cohort, a single 360° MLT session resulted in a modest but statistically significant IOP reduction of approximately 2.6 mmHg (14.2%) from a baseline mean of 18.15 mmHg. This effect was confirmed using a generalized estimating equation (GEE) model that accounted for inter-eye correlation, repeated measurements, and demographic covariates. The model revealed no significant influence of age, sex, or eye laterality on treatment response (*p* > 0.05 G for all), reinforcing the consistency of the effect across patient subgroups. Furthermore, interaction terms between treatment and both sex and age were tested and found to be non-significant, indicating that the IOP-lowering effect of MLT did not differ meaningfully by demographic profile. This degree of pressure lowering is clinically relevant, particularly in a pretreated population where most patients were already receiving multiple topical agents. It is well recognized that the efficacy of trabeculoplasty is inversely related to baseline IOP: eyes with higher initial values tend to show greater reductions, whereas those closer to target levels may experience smaller, but still meaningful, decreases [12]. This ceiling effect reflects the finite physiological capacity of the trabecular meshwork to increase aqueous outflow once near-optimal homeostasis is achieved. Consistent with this, prior studies reported IOP reductions between 18% and 22% during the first 6–12 months post-MLT, confirming the reproducibility of outcomes under real-world conditions [22,23,24].

Beyond mean IOP reduction, our analysis showed that 76.5% of treated eyes reached an IOP ≤ 18 mmHg at follow-up, compared to 50.8% at baseline. Similarly, 31.1% of eyes achieved a ≥20% IOP reduction, and 31.8% experienced a decrease of more than 3 mmHg, thresholds frequently regarded as clinically meaningful in real-world glaucoma care. These outcomes align with previously published MLT studies, where responder rates vary based on disease stage, prior therapy, and baseline IOP [12,25].

The pre- and post-treatment IOP distributions also indicated a shift toward tighter clustering within target ranges and a visible reduction in high-IOP outliers. This is important, as eyes with higher IOP variability or persistently elevated pressures, even within the high-teen range, remain at increased risk of progression, as highlighted in recent research on IOP dynamics and glaucoma risk [26].

The demographic and clinical composition of our cohort is like many glaucoma clinics: predominantly POAG (64.4%), with smaller proportions of pigmentary (14.4%) and pseudoexfoliative glaucomas, and a majority of pseudophakic eyes. This distribution enhances the external validity of our findings and is consistent with other MLT studies reporting similar age and sex distributions in the mid-60s, reflecting the epidemiology of chronic open-angle disease in aging populations [14,15]. The lack of association between lens status and IOP response in our series shows that MLT efficacy is independent of pseudophakia or prior cataract surgery, consistent with prior evidence from multicenter cohorts [25].

Gonioscopy assessment revealed wide-open angles (Shaffer grade 3–4) in most eyes, with one-third presenting anatomical variations such as heavy trabecular pigmentation, Sampaolesi lines, or focal synechiae. These features did not compromise treatment response, indicating that MLT maintains efficacy even in eyes with moderate anatomic heterogeneity. This observation aligns with data showing stable IOP reduction regardless of trabecular pigmentation intensity [17].

About one-third of patients presented ocular comorbidities such as pseudophakia, mild diabetic retinopathy, or dry age-related macular degeneration, none of which influenced MLT safety or efficacy, corroborating prior findings that the procedure can be safely performed in eyes with concurrent ocular pathology [12]. Taken together with the GEE findings, these convergent data indicate that the IOP-lowering effect of MLT in our cohort is unlikely to be an artifact of measurement method or statistical approach but rather a consistent treatment signal at both the eye and patient level [27,28].

Standardization of laser parameters is an important methodological strength of this study. Nearly all eyes underwent 360° MLT using a 300 µm spot, 300 ms exposure, 15% duty cycle, and 1000 mW power, settings concordant with those employed in controlled trials [15,29]. The use of full-angle application likely contributed to the uniform response, minimizing sectoral variability within the trabecular meshwork. The subthreshold micropulse mechanism, defined by microsecond pulses separated by thermal relaxation intervals, achieves biological stimulation without tissue destruction, explaining the absence of inflammatory reaction or peripheral anterior synechiae in our cohort. This biophysical mechanism differentiates MLT from earlier ALT and SLT approaches. ALT uses continuous-wave thermal energy that produces coagulative damage and trabecular scarring, limiting repeatability. In contrast, SLT employs selective photothermolysis and has been shown not to induce significant structural damage, although mild and transient cellular stress responses may occur [30,31].

In our study, this standardized protocol was applied over 360° of the trabecular meshwork in almost all eyes, with only a very small minority receiving sectoral treatment because of focal synechiae or suboptimal angle visualization. Although these few sectoral cases are too rare to permit formal subgroup analysis, the overall homogeneity of IOP reduction suggests that complete 360° application is both feasible and effective in routine practice, in agreement with recent prospective studies that have also favored full-angle MLT delivery [13,16]. Previous research reported that 360° 577 nm MLT at either 1000 or 1500 mW produced comparable short-term IOP reductions without compromising corneal structure [13], while another confirmed sustained pressure-lowering over 35 months with a 360° protocol in glaucoma and ocular hypertension [16]. These convergent data support the choice of circumferential treatment as a reproducible real-world strategy, reserving more limited sectors for eyes with anatomical constraints.

No patient in our cohort experienced an acute IOP spike > 5 mmHg. The laser’s on–off pulse pattern allows sufficient thermal relaxation between bursts, preventing cumulative energy buildup and minimizing cytokine-mediated trabecular dysfunction [14,18]. Moreover, comparative analyses have demonstrated that MLT results in fewer postoperative spikes, less discomfort, and comparable long-term pressure control [32,33]. In our series, only 3.8% of eyes showed a transient rise of ≤4 mmHg within the first hour, resolving without treatment, findings that were clinically mild and not associated with vision loss.

Visual and refractive stability throughout follow-up further supports the atraumatic nature of MLT. The absence of visual acuity loss or refractive change indicates that subthreshold photostimulation preserves the integrity of the cornea and anterior segment. These findings are in line with prospective imaging studies confirming that MLT does not affect endothelial cell density, corneal thickness, or anterior chamber morphology [18]. Specifically, BCVA remained unchanged (*p* = 0.553), reinforcing that the pressure reduction did not come at the expense of visual function. Regarding refraction, most categories were stable; however, the prevalence of hyperopic astigmatism decreased from 12.9% to 3.0% (*p* = 0.003). While statistically significant, this effect size is small and likely of limited clinical relevance, potentially reflecting minor corneal biomechanical changes after IOP lowering. The small reduction in hyperopic astigmatism observed in our series was statistically but not clinically significant and may reflect minor corneal biomechanical adjustments after IOP normalization [34]. This visual stability supports the use of MLT in outpatient settings without postoperative functional limitations. Recent work using dynamic Scheimpflug imaging and indentation devices has shown that pharmacologic or laser-induced IOP reductions can be accompanied by modest decreases in corneal stiffness and modulus without changes in central corneal thickness, particularly in eyes with glaucoma or ocular hypertension [34,35]. The small shift in hyperopic astigmatism in our cohort is consistent with these subtle biomechanical adjustments and supports the interpretation that MLT may slightly modulate the anterior segment biomechanical environment without inducing clinically relevant refractive change [36].

The safety profile in this study was very good. Only mild, transient symptoms such as conjunctival hyperemia (12%) or ocular discomfort (10%) occurred, resolving spontaneously or with short-term NSAID therapy. No cases of peripheral anterior synechiae, corneal edema, hypotony, or visual loss were recorded. These outcomes corroborate large-scale analyses reporting adverse event rates below 5% [37]. The low rate of inflammatory sequelae underscores the non-destructive, photobiomodulatory nature of MLT. Taken together with the non-significant changes in visual acuity and the absence of sight-threatening events, these observations underscore the favorable benefit–risk profile of MLT in routine care.

Our results suggest that the pre-existing medication load did not appear to limit the IOP-lowering effect of MLT, supporting its potential role as an adjunctive option in patients already receiving maximal tolerated therapy. Previous studies have shown that eyes on two or more agents still achieve an additional 15–20% IOP reduction after MLT [13], supporting its value in reducing polypharmacy and improving adherence in elderly or ocular surface–compromised individuals. This may position MLT as a useful approach to reduce chronic medication exposure while maintaining therapeutic control.

Another practical advantage of MLT is its repeatability. Because it induces no coagulative scarring, retreatment can be safely performed when IOP begins to rise again. Long-term studies have shown sustained effects for up to three years, though gradual attenuation may occur [15,16]. Randomized controlled trials using similar parameters (1000–1500 mW, 15% duty cycle) demonstrated treatment success (≥20% IOP reduction) in up to 75–80% of eyes at six months, with no endothelial loss or corneal edema [13].

Finally, the absence of significant postoperative complications or visual function loss in this real-world cohort supports the safety advantages of the micropulse approach. Combined with stable anterior segment anatomy and rapid recovery, these findings suggest that MLT may be a suitable outpatient option, particularly for elderly or multimorbid patients who may have limited tolerance for long-term medical therapy. In a cohort study of 296 eyes undergoing micropulse laser trabeculoplasty, no treatment-related complications were reported, and visual outcomes remained stable [37].

The findings support considering MLT as part of the therapeutic spectrum for glaucoma management, particularly in selected patient groups. For patients on maximal tolerated topical therapy, MLT offers a means of further pressure reduction without the added pharmacologic burden. This advantage is particularly relevant in elderly patients with ocular surface disease or those with poor adherence to multiple drops [8]. In early-stage glaucoma or ocular hypertension, MLT can serve as a primary intervention, delaying or potentially avoiding the need for chronic medications. This approach aligns with the emerging “laser-first” paradigm supported by current consensus. However, while MLT can postpone escalation toward surgical interventions, it should not be viewed as a substitute for incisional procedures when larger or long-term pressure reductions are required. Instead, it functions as a bridge within the therapeutic continuum, offering a minimally invasive option that can defer, but not replace, more invasive surgery in appropriately selected patients. Its mechanism of subthreshold photostimulation, rather than photodisruption, minimizes collateral damage, allows for safe retreatment, and avoids the need for postoperative corticosteroids [13].

In comparative studies, MLT and SLT achieve similar long-term efficacy, although SLT may induce slightly greater short-term reductions [14]. Data from multicenter registries also indicate non-inferiority between MLT and SLT at 12 months in terms of absolute IOP reduction (17%) and medication reduction, with MLT producing fewer transient pressure spikes and virtually no structural damage to the trabecular meshwork [37].

Although MLT is not traditionally considered a first-line option in advanced glaucoma, its favorable safety and tolerability profile may justify its use in carefully selected high-risk patients. When positioned within the broader surgical spectrum, MLT remains the least invasive and most conjunctiva-sparing procedure available, preserving the ocular surface for potential future interventions such as minimally invasive glaucoma surgery or filtration procedures. Its minimal recovery time, repeatability, and excellent tolerability make it an attractive option in health systems where access to surgery is limited or for patients who wish to postpone or temporarily avoid incisional operations, although it does not replace surgery when more substantial or sustained pressure lowering is required. In this regard, MLT complements both medical and surgical management, functioning as a flexible tool that can be tailored to disease severity and patient needs.

The main strengths of this study lie in its real-world design, standardized treatment parameters, and comprehensive assessment of outcomes using dual tonometry and visual function measures. The use of a uniform 360° protocol (577 nm, 300 µm, 300 ms, 15% duty cycle, 1000 mW) across all eyes ensures reproducibility and comparability with contemporary studies, while inclusion of various open-angle subtypes enhances external validity. The cohort reflects a typical clinical spectrum of glaucoma, including medicated and pseudophakic patients, demonstrating that MLT performs consistently under routine conditions. The absence of significant adverse events or IOP spikes further supports the excellent safety profile of the technique and confirms that subthreshold photostimulation can be applied safely across different pigmentary and anatomical contexts.

Nonetheless, several limitations should be acknowledged. The lack of a randomized control group and the observational design limit direct comparison with SLT or medical therapy. Follow-up duration varied among patients, precluding precise evaluation of long-term durability, and medication reduction was not formally quantified. Although the sample size was adequate to detect significant IOP changes, larger multicenter studies would improve statistical power and generalizability. While the primary analysis used a GEE model adjusted for age, sex, and eye laterality, no significant interactions were found between treatment effect and these covariates. However, the study was not powered to detect subtle subgroup differences, and future studies with larger samples may benefit from expanded multivariable GEE or mixed-effects models. These approaches could explore additional baseline predictors of treatment response and align with recent biostatistical guidance in ophthalmic research, where covariate-adjusted longitudinal models are increasingly recommended [27]. Baseline IOP values already in the high teens likely attenuated the relative reduction achievable, reflecting the physiological ceiling for trabecular interventions in pretreated eyes. Finally, while structural and molecular imaging were beyond the scope of this analysis, future research integrating anterior segment OCT or aqueous cytokine profiling could clarify the mechanisms underlying the trabecular remodeling response to MLT.

Despite these limitations, the consistent efficacy, safety, and tolerability indicated here strengthen the evidence supporting MLT as a reproducible, minimally invasive option within the evolving therapeutic continuum of glaucoma management.

## 5. Conclusions

Micropulse laser trabeculoplasty led to a modest but significant IOP reduction after a single treatment session, with stable visual acuity and no major complications, supporting its favorable safety profile. Its non-destructive nature and good tolerability make it a suitable adjunct for patients needing additional pressure control or deferral of surgery.

However, the lack of a control group limits causal interpretation and comparison with other treatments. While the design and analysis strengthen internal validity, larger controlled studies are mandatory for confirming these findings and clarifying MLT’s role in glaucoma management.

## Figures and Tables

**Table 1:** Standard parameters used for Micropulse Laser Trabeculoplasty (MLT).

Parameter	Setting	Notes
Laser wavelength	577 nm	Diode-pumped solid-state laser (Iridex system)
Spot size	300 µm (200 µm in selected cases)	Adjusted according to trabecular visualization
Exposure duration	300 ms	Each laser application
Duty cycle	15%	Corresponding to micropulse on/off energy cycles
Power	1000 mW	Slightly adjusted depending on trabecular pigmentation
Treatment extent	360° (90–300° in selected cases)	Full or partial coverage of the trabecular meshwork

**Table 2:** Standardized ophthalmologic evaluations before and after MLT.

Assessment	Device/Method	Description/Purpose
Visual acuity	Snellen chart	Best-corrected visual acuity (BCVA)
Refraction	Autorefractometer	Objective refraction
Slit lamp and fundus exam	Standard slit-lamp	Evaluation of anterior and posterior segments
Gonioscopy	Gonioscopy lens (Ocular Instruments, Bellevue, WA, USA)	Shaffer grading (0–4), documentation of trabecular pigmentation, Sampaolesi line, synechiae, pseudoexfoliation material
Pachymetry	Optopol Revo NX (Optopol Technology, Zawiercie, Poland)	Measurement of corneal thickness (µm)
Intraocular pressure (IOP)	Goldmann applanation tonometer	Reference method for IOP measurement. Values were recorded without pachymetric correction and used for all analyses.
OCT—anterior segment and optic nerve	Optopol Revo NX	Analysis of angle configuration and ganglion cell structure
Computerized perimetry	Optopol PTS 2000 (Optopol Technology, Zawiercie, Poland)	Functional correlation between visual field and structural OCT data

**Table 3:** Baseline demographic profile and eye laterality of included patients.

Parameter	Category/Range	n	%
Age (years)	<50	21	15.9
	51–60	20	15.2
	61–70	52	39.4
	71–80	24	18.2
	>80	15	11.4
	Mean ± SD (range)	63.99 ± 12.88 (27–87)	
Gender	Female	83	62.9
	Male	49	37.1
Laterality	Right eye (RE)	13	16.3
	Left eye (LE)	15	18.7
	Both eyes (RE + LE)	52	65.0
	Total OD	65	49.2
	Total OS	67	50.8
	Total eyes	132	100.0

**Table 4:** Clinical and gonioscopic characteristics of eyes treated with MLT.

Parameter	Category/Range	n	%
Diagnosis	Primary open-angle glaucoma (POAG)	85	64.4
	Advanced POAG	17	12.9
	Pigmentary glaucoma	19	14.4
	Pseudoexfoliative glaucoma	4	3.0
	Secondary glaucoma	5	3.8
	Late juvenile glaucoma	2	1.5
Gonioscopic configuration (Shaffer grade)	Grade 2	15	11.4
	Grade 3	21	15.9
	Grade 4 (widest open angle)	96	72.7
Gonioscopic particularities	None observed	89	67.4
	Any particularity	43	32.6
	Heavily pigmented trabecular meshwork	26	60.5
	Sampaolesi line	7	16.3
	Peripheral anterior synechiae (focal)	6	14.0
	Hypopigmented trabeculae	4	9.3
	Pseudoexfoliative deposits	4	9.3
	High iris root/posterior insertion	2	4.7
	Blood in Schlemm’s canal	2	4.7

**Table 5:** Distribution of cases according to associated ophthalmologic pathology.

Associated Ophthalmologic Pathology	n	%
None	84	63.6
Any comorbidity	48	36.4
Pseudophakia	37	77.1
Age-related macular degeneration (dry type)	7	14.6
Cataract	3	6.3
Diabetic retinopathy	3	6.3
Interstitial keratitis	4	8.3
Retinal detachment	1	2.1
Macular hole	2	4.2
Glaucoma microstent	2	4.2
Express shunt	3	6.3

**Table 6:** Distribution of eyes according to ocular treatment.

Treatment Class	Eyes	
	n	%
Carbonic anhydrase inhibitors (Dorzolamide/Brinzolamide)	81	83.5
Prostaglandin analogs	73	75.3
Beta-blockers	69	71.1
Fixed combinations	69	71.1
Alpha-agonists (Brimonidine)	24	24.7
Acetazolamide	7	7.2

**Table 7:** GEE model output for IOP reduction after MLT.

Effect	Coefficient	Std. Error	z	*p* Value	95% CI (Lower)	95% CI (Upper)
Intercept (post)	18.94	0.82	23.10	<0.001	17.33	20.54
Moment (pre- vs. post-)	2.58	0.47	5.48	<0.001	1.66	3.51
Sex (M vs. F)	−1.39	1.11	−1.25	0.210	−3.56	0.78
Age	−0.023	0.035	−0.67	0.505	−0.091	0.045
Eye (OD Vs. OS)	−0.244	0.418	−0.58	0.559	−1.064	0.576
Moment × sex	−0.001	0.721	−0.002	0.999	−1.414	1.412
Moment × age	0.031	0.022	1.43	0.153	−0.012	0.075

**Table 8:** Clinical outcomes before and after MLT.

Clinical Outcome	Pre-Treatment	Post-Treatment	Statistical Test
Eyes with IOP ≤ 18 mmHg	67/132	101/132	McNemar exact,
	(50.80%)	(76.50%)	*p* = 0.0014
Eyes with IOP ≤ 21 mmHg	114/132	126/132	McNemar exact,
	(86.30%)	(95.40%)	*p* = 0.0023
Eyes with ≥20% IOP reduction	—	41/132 (31.1%)	—
Eyes with IOP decrease > 3 mmHg	—	42/132 (31.8%)	—

**Table 9:** Visual acuity and refractive outcomes before and after MLT.

Parameter	Pre-Treatment	Post-Treatment	Statistical Result
Best-Corrected Visual Acuity (BCVA)			
Mean ± SD (decimal fraction)	0.67 ± 0.33	0.70 ± 0.33	F = 0.353; 95% CI: 0.65–0.73; *p* = 0.553
Refraction			
Emmetropia	2 (1.5%)	1 (0.8%)	—
Myopia	56 (42.4%)	54 (40.9%)	χ^2^ = 0.061; 95% CI: −10.26–12.20%; *p* = 0.805
Hyperopia	74 (56.1%)	77 (58.3%)	χ^2^ = 0.130; 95% CI: −9.61–13.93%; *p* = 0.718
Hyperopic astigmatism	17 (12.9%)	4 (3.0%)	χ^2^ = 8.806; 95% CI: 3.41–16.93%; *p* = 0.003
Myopic astigmatism	90 (68.2%)	94 (71.2%)	χ^2^ = 0.280; 95% CI: −8.03–13.93%; *p* = 0.596
Mean cylindrical axis (°) ± SD	73.00 ± 52.60	75.01 ± 53.79	t = 0.307; 95% CI: −10.88–14.90; *p* = 0.759

**Table 10:** Adverse events and postoperative findings after MLT.

Adverse Event	Treated Eyes n (%)	Severity/Duration	Management/Outcome
Mild ocular discomfort during laser	13 (9.8)	Transient	None required
Mild conjunctival hyperemia/ache	16 (12.1)	1–2 days	Resolved spontaneously or with topical NSAIDs
Mild anterior chamber cells and flare	9 (6.8)	≤1 week	Resolved with NSAID drops
Transient IOP increase (≤4 mmHg)	5 (3.8)	Within 1 h post-MLT	Normalized without intervention

## Data Availability

All data are contained within the article.

## Abbreviations

The following abbreviations are used in this manuscript:

AC	anterior chamber
ALT	argon laser trabeculoplasty
AMD	age-related macular degeneration
ANOVA	analysis of variance
BCVA	best-corrected visual acuity
CCT	central corneal thickness
CI	confidence interval
DC	duty cycle
DR	diabetic retinopathy
ECM	extracellular matrix
GAT	goldmann applanation tonometry
IL-1β	interleukin-1 beta
IOP	intraocular pressure
LE	left eye
MLT	micropulse laser trabeculoplasty
Nd:YAG	neodymium-doped yttrium aluminum garnet
NCT	non-contact tonometry
NSAID	non-steroidal anti-inflammatory drug
OAG	open-angle glaucoma
OHT	ocular hypertension
OCT	optical coherence tomography
OD	oculus dexter (right eye)
OS	oculus sinister (left eye)
PAS	peripheral anterior synechiae
POAG	primary open-angle glaucoma
RE	right eye
RGC	retinal ganglion cell
SD	standard deviation
SLT	selective laser trabeculoplasty
TM	trabecular meshwork
TNF-α	tumor necrosis factor alpha
VA	visual acuity
